# Stability and Antioxidant Activity of *Pouteria macrophylla* Fruit Extract, a Natural Source of Gallic Acid

**DOI:** 10.3390/molecules28083477

**Published:** 2023-04-14

**Authors:** Raioní K. Pantoja, Camila Fernanda B. Albuquerque, Rafael A. do Nascimento, Lênio José G. De Faria, José Guilherme S. Maia, William N. Setzer, Tais Gratieri, Joyce Kelly R. da Silva

**Affiliations:** 1 Laboratório de Biotecnologia de Enzimas e Biotransformações, Universidade Federal do Pará, Belém 66075-110, PA, Brazil; raonikempfer@hotmail.com (R.K.P.); camila.barbosa@icb.ufpa.br (C.F.B.A.); 2 Laboratório de Engenharia de Produtos Naturais, Universidade Federal do Pará, Belém 66075-110, PA, Brazil; rafaelnascimentoa@gmail.com (R.A.d.N.); lenio@ufpa.br (L.J.G.D.F.); 3 Programa de Pós-Graduação em Química, Universidade Federal do Maranhão, São Luís 65080-805, MA, Brazil; gmaia@ufpa.br; 4 Aromatic Plant Research Center, Suite 100, Lehi, UT 84043, USA; wsetzer@aromaticplant.org; 5 Laboratory of Food, Drug and Cosmetics (LTMAC), School of Health Sciences, University of Brasilia, Brasília 70910-900, DF, Brazil; tgratieri@gmail.com

**Keywords:** cutite, phenolic compounds, Amazon fruits, surface methodology, Box and Behnken experimental design

## Abstract

*Pouteria macrophylla* (cutite) fruits are rich in phenolic acids, resulting in antioxidant and skin depigmenting activity. The aim of this study, then, is to evaluate the cutite extract stability under three variations of light, time, and temperature using a Box–Behnken experimental design to analyze through the surface response the variations of the total phenolic content (TPC), antioxidant activity (AA), and gallic acid content (GA). A colorimetric assay was also performed, and a decrease in the darkening index was noticed due to the high phenolic coloration in the presence of light, indicating less degradation to extract stability. The experimental planning showed variations in all responses, and second-order polynomial models were calculated and considered predictable, as well as the effects were significant. The TPC exhibited a variation in less concentrated samples (0.5% p/v) at higher temperatures (90 °C). In contrast, the temperature was the only influential variable for AA, where only higher temperatures (60–90 °C) were able to destabilize the fruit extract. Differently, GA showed only the concentration as the influential variable, exhibiting that neither temperature nor time of exposure could affect the gallic acid content stability of *P. macrophylla* extract. For this, *P. macrophylla* extract was shown to be highly stable, providing a great perspective on cosmetic application.

## 1. Introduction

The Sapotaceae family has 53 genera and about 1250 species, being found globally but mainly in Asia and South America’s tropical and subtropical regions [1]. The *Pouteria* genus, in turn, is tropical, composed of 325 species, and characterized by having a significant economic value due to high-quality wood, edible fruits, and its growing use in popular medicine for several purposes [2,3].

The chemical composition of the genus consists mainly of triterpenes and flavonoids, some of which are found regularly in several species in different tissues as leaves, barks, flowers, and fruits [4]. Long-chain hydrocarbons, alcohols, phenolic acids, fatty acids, and esters are also present, and some species are also sources of enzymes and structures of great biotechnological value, such as polyphenol oxidases (POP) [3,5]. Among the species of the genus, we can mention extracts from the fruits of *P. caimito, P. cambodiana, P. campechiana, P. sapota, P. torta*, and *P. viridis* as presenting potential antimicrobials, cytotoxicity, anti-inflammatory, and antidiabetics. In addition, the antioxidant activity attributed to the presence of phenolic compounds, lupeol, ursolic acid, carotenoids, and other triterpenes has been reported. [3,6,7,8,9,10,11,12,13,14,15,16,17]. 

**Figure 1:**
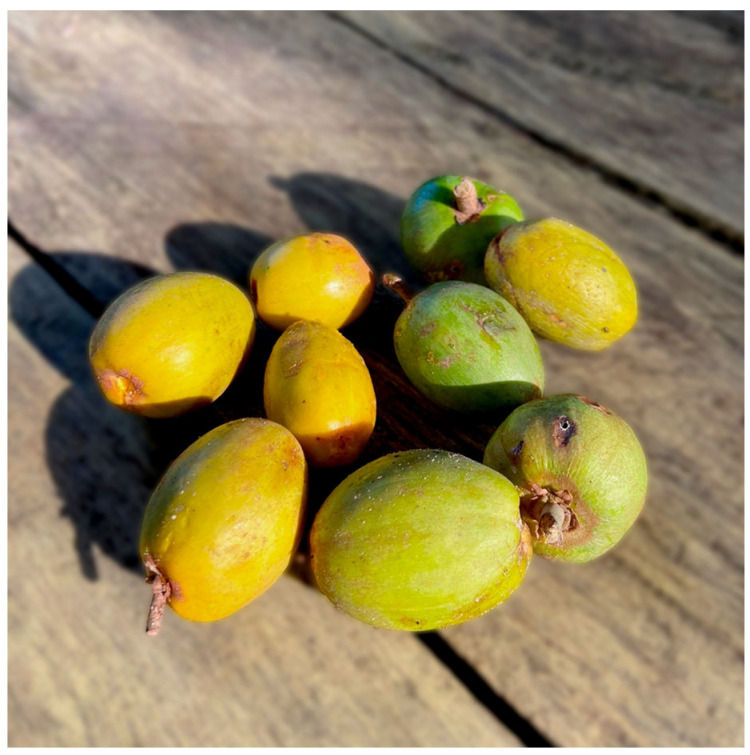
*Pouteria macrophylla* fruit (source: author).

The fruit of *Pouteria macrophylla* (Figure 1) has a spherical shape, starchy, with a characteristic sweet taste, yellowish-green peel, and yellowish flesh, with one or two seeds on average. Its most frequent application is consumption in food, where the fruit is generally in an advanced stage of ripeness [18]. In addition, studies related to this species show important biological roles, such as antioxidant actions and skin depigmentation activity, suggesting a possible use of these characteristics for health and human well-being [19,20,21]. 

The chemical composition of *P. macrophylla* ethanolic fruits extract has been reported previously for our research group and comprises high values of gallic acid (13.4 mg/g DW) and quercetin (0.044 mg/g DW) [19]. Other phenolic acids were also identified in aqueous extracts of *P. macrophylla* fruit, such as *p*-cumaric acid (0.0276 mg/g DW), vanillinic acid (0.0055 mg/g DW), ferulic acid (0.0047 mg/g DW), 3,4-dihydroxybenzoic (0.0046 mg/g DW), synaptic acid (0.0045 mg/g DW), caffeic acid (0.0031 mg/g DW), epicatechin (0.0025 mg/g DW), chlorogenic acid (0.0009 mg/g DW), catechin (0.0006 mg/g DW), and rutin (0.00022 mg/g DW) [21].

The chemical characterization and antioxidant activity of the aqueous extract of the *P. macrophylla* fruit by HPLC-MS (high-performance liquid chromatography-mass spectrometry), 2,2-diphenyl-1-picrylhydrazyl (DPPH) assay, and the Folin–Ciocalteu method indicated a high concentration of total phenolic compounds and antioxidant activity, mainly attributed to the high content of gallic acid (GA) (12.47 mg/g dry mass). Additionally, when assayed by the TOSC method, the fruit extract displayed a high capacity to scavenge two important ROS (reactive oxygen species): the radicals peroxyl and peroxynitrite [21].

Microemulsions with depigmenting action containing ethanolic extracts of *P. macrophylla* fruits were developed to be nonirritating in a reconstructed human epidermis model in vitro, confirming its safety [19]. GA is present in different vegetable families and has been used as an active ingredient in the industry thanks to its anti-inflammatory, antifungal, antiviral, antibacterial, antitumor, antioxidant, and non-allergenic action, in addition to its potential for preventing aging [22,23,24]. Antioxidants are substances widely used to prevent oxidative stress. In addition to the human body being equipped with a defense system capable of generating endogenous antioxidants, the body can incorporate these substances through food or topical use, reducing the effect of free radicals [25]. However, the physicochemical environment, such as light, oxygen, pH, and darkness, of their storage provokes significant variations in their antioxidant activity [26]. In this sense, for a potential industrial application, it is imperative that both the antioxidant activity and stability of the components are maintained during the whole manufacturing process, i.e., from the extract procedure to the formulation incorporation. Therefore, responses to these must be anticipated. 

Thus, the aim of this work was to evaluate the stability of the fruit extract of *P. macrophylla* through variations of light and temperature and its influence on the antioxidant activity and the total phenolic content, aiming for potential industrial applications.

## 2. Results

### 2.1. Evaluation of Colorimetric Assay

**Figure 2:**
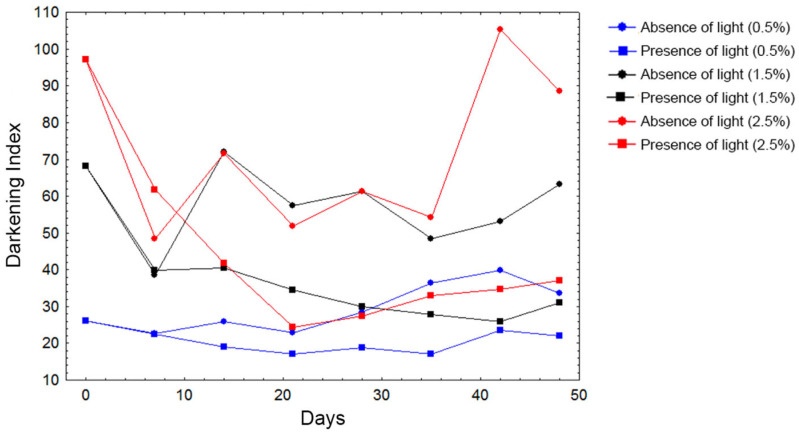
Darkening index related to 0.5%, 1.5%, and 2.5% (*m*/*v*) of fruit in ethanolic extracts throughout the following days in chamber in the presence or absence of light. The colors (blue, black, and red) represent the concentrations, while the forms (circles or squares) indicate the luminosity.

Throughout seven weeks, the color of the samples was evaluated using a colorimeter. It was noticed that the samples stocked in illuminated chambers showed a decrease in the darkening index, as shown in Figure 2. 

### 2.2. Evaluation of Experimental Planning

Purposeful modifications of the levels (−1, 0, and 1) of the PBB resulted in variations of the TPC content (10.83–21.04 mg EAG/g), AA (61.68–133.25 µg TE/g), and GA (25.36–251.25 mg GAE/g) (Table 1), indicating, at first, that the operational variables influenced the response. By the multiple regression analysis performed on the experimental data, the predicted responses could be obtained via the second-order polynomial equation, which regression coefficient values are shown in Table 1.

The coded variables X1, X2, and X3 could be related to the real variables (Tp, Tr, and C, respectively). The generated polynomial models presented satisfactory coefficients of determination for biological processes, indicating a percentage ≥75.60% in describing the experimental variability around the average. The models followed the statistical assumptions of normality, homoscedasticity, and independence of residuals (not included in the article), and the lack of adjustment was not significant for any of the answers (*p* ≥ 0.07), indicating the adequacy of the predictions of the second-order polynomial models. 

**Figure 3:**
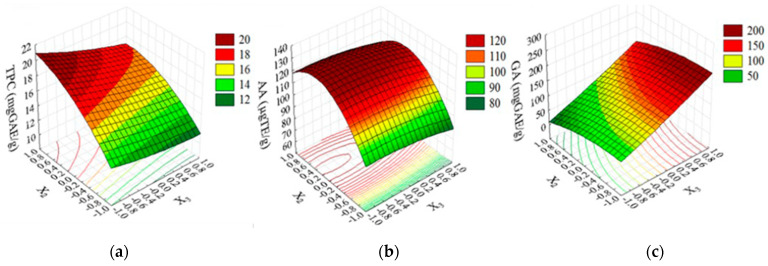
Response surface for the TPC (**a**), AA (**b**), and GA (**c**) at X1= −1.

Through ANOVA (Table 2), it was identified which operational variables and their combinations exerted a significant influence (*p* ≤ 0.05) in each of the responses. The construction of the response surface (Figure 3) allowed the visualization of how changes in operational variables influenced the magnitudes of the TPC, AA, and GA, as well as the stability of the extracts in each condition. As the contact time (X_1_) was the only statistically noninfluential variable (Table 3) in all responses, the 3D graph was constructed at X_1_ = −1 to make the process cheaper.

### 2.3. Influence of Operational Variables on TPC, AA, and GA

The polynomial model for the total phenolic content was obtained by multiple regressions, where the coded variables X_1_, X_2_, and X_3_ can be related to real variable times, temperatures, and concentrations, respectively, as shown below:

TPC = 13.78 − 0.55X_1_ + 1.50X_2_ − 1.22X_3_ + 2.36X_1_^2^ − 1.41X_2_^2^ + 0.29X_3_^2^ − 1.90X_1_X_2_ + 0.16X_1_ X_3_ − 0.55X_2_X_3_ (1)

The TPC was significantly affected (*p* ≤ 0.047) only by the linear terms temperature (X_2_) and concentration (X_3_), as shown in the ANOVA (Table 3). High TPC values were obtained at a higher temperature (X_2_ = 1.0; Tr = 90 °C) and lower concentration of *P. macrophylla* extract (X_3_ = −1.0; C = 0.5%).

Equation (2) describes the variations in antioxidant activity (µgTE.g^−1^) of ethanolic cutite extracts.

AA = 119.91 − 5.77X_1_ + 15.44X_2_ − 4.12X_3_ + 6.88X_1_^2^ − 23.21X_2_^2^ + 2.93X_3_^2^ − 10.84X_1_X_2_ + 0.11X_1_X_3_ − 3.45X_2_X_3_ (2)

The temperature, either in linear or quadratic form, was the only statistically influential variable on AA (*p* ≤ 0.047). The regression coefficient shown in Table 2 indicates a positive influence of temperature on the AA. This correlation is exemplified in Figure 3b, where the highest AA values (120–140 µg TE/g) were obtained at 60 to 90 °C (X2 = 0 to 1.0). 

Equation (3), instead, describes the influence of operational variables on the gallic acid content in cutite ethanolic extracts:

GA = 143.88 + 4.67X_1_ − 26.53X_2_ + 70.08X_3_ − 23.27X_1_^2^ − 14.58X_2_^2^ + 5.05X_3_^2^ − 9.22X_1_X_2_ + 24.93X_1_X_3_ − 18.88X_2_X_3_ (3)

The concentration of fruit extract (X_3_) was the only statistically influential variable (*p* = 0.029) on the GA content, which highest GA values (~200 mg EAG/g) were observed at X_3_ = 1 (Concentration = 2.5%), regardless of any temperature. 

## 3. Discussion

Recently, the cosmetic potential of *P. macrophylla* fruits extract has been reported as a skin depigmentation agent [19]. However, studies identifying the impact of storage conditions on antioxidant activity are still absent. In the literature, only a few studies are based on quality control during Pouteria fruit storage. The results observed in *P. campechiana* fruits showed that the storage time significantly affected the amounts of carotenoids, tannin, phenolic, and flavonoids. The highest antioxidant activity of the fruit extracts was obtained after 10 days of storage [27]. This feature, which is determined mainly by phenolic content stability and gallic acid content specifically in this fruit, is imperative for a successful industrial application. For this, the darkening index, total phenolic compounds, antioxidant activity, and gallic acid content were analyzed to quantify and characterize the stability of these fruit extracts and, hence, their application as a cosmetic formulation ingredient.

The colorimetric assay showed a decrease of the darkening index, which means that the samples became clearer in the presence of light throughout the days. The yellow color of several *Pouteria* fruits was attributed to the presence of carotenoids, which present a high unsaturation rate [4]. Factors such as heat and light can promote the *trans*-isomerization of carotenoids, which are more stable as a *cis*-form, promoting a slight loss of color and provitamin activity [28]. Other studies searching the photodegradation of phenolic-colored extracts also noticed this same behavior in high phenolic-colored extracts, addressing less degradation to the solution stability, mostly because of the interaction between the phenolic compounds and the optimal pH of the samples [29,30,31].

The influence of the variables in the total phenolic content showed a proportional influence of temperature (X_2_ = +1) and an inversely proportional influence of concentration (X_3_ = −1) in the TPC response. When the fruit concentration was lower and the temperature was higher, the TPC was approximately 20 mg EAG.g^−1^, as shown in the 3D response surface graph (Figure 3a). In the literature, the value of the degradation temperature of phenolic compounds in fruit extracts is controversial. It was previously described that the processing and storage of vegetables directly influences the retainment of phenolic compounds after extraction, which means that better treatment methodologies result in a higher retention of phenolic acids [32]. Due to this, there is some divergent data about the stability of TPC in fruit extracts. 

Temperatures over 60 °C can cause the minor degradation of gallic acid in an aqueous extract of grapes; it was also noticed that polyphenols of white onions and its roots can still be stable (38.0 and 40.0%, respectively) even after 60 min at boiling temperature (>100 °C). The standard solutions of GA were exposed for 4 h to high temperatures and showed relative stability. Gallic acid displayed a higher antioxidant activity, and the results showed that, even after 3 h of UV-C exposure, approximately 50% of gallic acid was removed. Higher concentrations of phenolic compounds and the complexity of chemical compositions in fruit extracts can explain the stability of its compounds [33,34]. A hypothesis for the paradoxical results found in our studies is that the higher experimental temperature (120 °C) caused ethanol to evaporate and the phenolic compounds to concentrate, increasing the TPC values and variating the stability of the phenolic compounds in *P. macrophylla* extract.

A positive influence by temperature was observed in the antioxidant activity in *P. macrophylla* extracts, and temperatures around 60 to 90 °C made it increase. Other studies showed the positive effects of thermal treatment on the DPPH radical scavenging activity of some extracts from various agricultural byproducts [35,36,37], possibly from the tendency of phenols to combine themselves through polymerization, exerting a higher antiradical activity than the original monomers [36,38]. As the expected storage temperatures of cosmetic formulations are lower, and temperatures below 60°C showed no influence on the stability, the results indicate an optimistic perspective.

The treatments did not display influence on the GA content. Thermal analyses of the gallic acid degradation showed that it is a heat-stable compound, with a melting point of 258–260 °C [39]. The first degradation ratio was noticed only in temperatures higher than 105 °C [40], corroborating the results found, where temperature or time of exposure do not influence the stability of the gallic acid content in *P. macrophylla* ethanolic extracts, suggesting that all potential biological applications will be maintained. 

## 4. Materials and Methods

### 4.1. Plant Material

*Pouteria macrophylla* (Lam.) Eyma fruits were collected from the metropolitan region of the city of Belém (PA, Brazil) in October/2018 and identified by comparison with authentic *P. macrophylla* vouchers from Museum Emílio Goeldi Herbarium (MG239766). For storage, about 600 g of the fruit were lyophilized and stored under refrigeration at −18 °C.

### 4.2. Assays

#### 4.2.1. Fruit Extracts

The lyophilized fruits were pulverized and dissolved in ethanol at three different proportions: 0.5%, 1.5%, and 2.5% of fruit mass per milliliter of ethanol. To assess the dissolution yield, the weight of the sample before the extraction was measured and centrifuged after adding the solvent, followed by a second weighing of the dry precipitate, reaching a value of 50%.

#### 4.2.2. Stability Study

The extracts were submitted to a stability study elaborated according to the experimental analysis method [41]. The analytical methods employed included physical–chemical assessments. Three concentrations of the extracts were placed in test tubes (5 mL), sealed, and stored in two different storage conditions: chamber at room temperature (around 25 °C) without exposure to light and chamber at the same temperature but with a constant incidence of artificial lighting (32 W, 6400 K). The parameters chosen for the periodic analyses were colorimetric evaluation, antioxidant activity, total phenolic compounds content, and gallic acid. The study was carried out for seven weeks, and the samples were analyzed weekly from the first day of storage.

#### 4.2.3. Colorimetric Assay

The colorimetric characteristic was evaluated with a colorimeter, the samples from the first week of the initial control, and the samples stored in a greenhouse without lighting as a second control. The color spaces and numerical values are used to develop, present, and visualize colors in two- or three-dimensional spaces. Color is usually measured in L*a*b* parameters, an international standard color space. The value of L* is expected to decrease as darkening occurs once the L* value indicates the luminance/luminance in an image, ranging from 0 to 100 (0 indicates black; 100 indicates white). The parameters a* (from green to red) and b* (from blue to yellow) are the two chromatic components that range from −120 to 120 [42,43]. Thus, the darkening index (IE) parameter was determined by the following equation:

IE = [100(x − 0.31)]/0.17,(4)

where

x = (a* + 1.75L*)/(6.645L* + a* − 3.012b*)(5)

#### 4.2.4. Total Phenolics

The total phenolics (TP) concentration in the ethyl acetate extracts was determined according to the Folin–Ciocalteu procedure [44,45]. The extracts were solubilized in methanol at an initial concentration of 20 mg.mL^−1^ and diluted with water. Aliquots (400 µL) of the aqueous solution were mixed with 250 µL of Folin–Ciocalteu reagent (1.0 N) and 1250 µL of sodium carbonate (75 g.L^−1^). The absorbance was measured after 30 min at 760 nm and 25 °C (UV–Vis spectrophotometer, Biosystems RA2708). The experimental calibration curve was prepared using gallic acid at concentrations of 0.0 to 8.0 mg.L^−1^, submitted for the same procedure. The total phenolics content was expressed as gallic acid equivalents (GAE) in milligrams per gram of extract (mg GAE.g^−1^). 

#### 4.2.5. Antioxidant Activity

The antioxidant activity of the extracts was evaluated by the DPPH radical scavenging method [46,47]. The extracts were solubilized in methanol (20 mg.mL^−1^). Aliquots of the solution (50 µL) were mixed with 1950 µL of DPPH solution. The absorbance was measured after 20 min at 517 nm, and the DPPH radical scavenging inhibition was calculated using this equation:

I(%) = [1 − (Asample/Acontrol)] × 100(6)

The total antioxidant capacity was expressed as the equivalent of Trolox (TEAC), calculated from a standard curve with the Trolox (6-hydroxy-2,5,7,8-tetramethyl chroman-2-carboxylic acid) at concentrations 0.25, 0.5, 0.75, 1.0, and 1.25 mg.mL^−1^.

#### 4.2.6. Analysis of Gallic Acid Content

The determination of the gallic acid content was carried out by reading the samples at 50× dilution in a spectrophotometer at 260 nm, the results being compared with a standard curve elaborated from the reading of samples of gallic acid dissolved in distilled water in the concentrations of 1, 2.5, 5, 10, 15, and 25 µg.mL^−1^. The gallic acid content of the samples, expressed in milligrams of gallic acid equivalent (GAE) per gram of the sample, was obtained by multiplying the absorbance by the dilution and angle of the standard curve (y = −27.84x; R² = 0.9996), followed by obtaining the average in triplicate.

#### 4.2.7. Statistical Analysis

The extracts were momentarily exposed to different temperatures at different times. The experiments were designed according to the experimental design of Box and Behnken (PBB) [48] for three factors: time of exposure (X_1_), the temperature of exposure (X_2_), and the extract concentration (X_3_), totaling fifteen experimental runs. The coded values of the input variables are shown in Table 1, and the contents of the total phenolics (TPC), antioxidant activity (AA), and gallic acid (GA) were evaluated as response variables.

The polynomial model generated by this type of planning is shown in Equation (7), which corresponds to each of the response variables: x is the coded level of the design variables; n is the number of operational variables; b_0_ is the intercept; and b_i_, b_ii_, and b_ij_ are the regression coefficients of the linear, quadratic, and linear interaction terms, respectively.

Y = b_0_ + ∑ b_i_X_i_ + ∑ b_ii_X_i_^2^ + ∑ b_ij_X_ij_ (7)

The PBB results were interpreted based on the analysis of variance (ANOVA) to identify which independent variables, alone or in combination, were significant in the process. The statistical models proposed for the responses were evaluated based on the coefficient of determination (R^2^) and residual analysis (not included in the article). All statistical analyses were performed considering a confidence level of 95% (*p* < 0.05), and the results were obtained using the software Statistica version 7.0.

## 5. Conclusions

The results exhibited in this study indicated that *Pouteria macrophylla* fruit extract had excellent antioxidant activity and a high total phenolic content, being stable for domestic storage conditions in terms of temperature and lighting. The antioxidant activity, even for the lowest concentration percentage, exhibited relevance. Therefore, the employment of cutite extract in cosmetic formulations is consistent and capable of providing great advantages to human health and beauty applications.

## Figures and Tables

**Table 1:** PBB experiment matrix and dependent variable responses.

Assay	Tm (X_1_)	Tp (X_2_)	C (X_3_)	TPC (mgGAE.g^−1^)	AA (µgTE.g^−1^)	GA (mgGAE.g^−1^)
1	60 (−1)	30 (−1)	1.5 (0)	11.27	72.44	102.34
2	120 (+1)	30 (−1)	1.5 (0)	12.21	79.58	89.28
3	60 (−1)	90 (+1)	1.5 (0)	21.04	133.25	141.22
4	120 (+1)	90 (+1)	1.5 (0)	14.38	97.03	91.29
5	60 (−1)	60 (0)	0.5 (−1)	17.41	119.16	49.94
6	120 (+1)	60 (0)	0.5 (−1)	17.75	110.40	50.25
7	60 (−1)	60 (0)	2.5 (+1)	14.79	121.10	151.21
8	120 (+1)	60 (0)	2.5 (+1)	15.76	112.78	251.25
9	90 (0)	30 (−1)	0.5 (−1)	13.40	87.23	114.14
10	90 (0)	90 (+1)	0.5 (−1)	14.51	102.95	25.36
11	90 (0)	30 (−1)	2.5 (+1)	11.91	61.68	281.11
12	90 (0)	90 (+1)	2.5 (+1)	10.83	91.22	116.79
13	90 (0)	60 (0)	1.5 (0)	13.03	100.62	105.26
14	90 (0)	60 (0)	1.5 (0)	14.59	118.25	172.72
15	90 (0)	60 (0)	1.5 (0)	13.73	116.85	153.67

Tm: time (min); Tp: temperature (°C); C: concentration (% *m*/*v*); TPC: total phenolic content (mg EAG.g^−1^); AA: antioxidant activity; GA: gallic acid content.

**Table 2:** Analysis of the variance and polynomial regression coefficients.

Factors	df	TPC			AA			GA		
		Coef	p	SS	Coef	p	SS	Coef	*p*	SS
X_1_	1	−0.550	0.184	2.421	−5.769	0.238	266.299	4.669	0.741	174.40
X_2_	1	1.496	0.032 *	17.896	15.438	0.047 *	1906.606	−26.528	0.164	5630.09
X_3_	1	−1.222	0.047 *	11.948	−4.119	0.357	135.744	70.083	0.029 *	39,292.83
X_1_^2^	1	2.355	0.028 *	20.482	6.881	0.310	174.813	−23.269	0.505	1999.27
X_2_^2^	1	−1.410	0.074	7.345	−23.211	0.045 *	1989.275	−14.581	0.1479	784.99
X_3_^2^	1	0.290	0.548	0.311	−2.926	0.624	31.604	5.047	0.806	94.06
X_1_X_2_	1	−1.900	0.039 *	14.445	−10.840	0.157	469.992	−9.217	0.649	339.82
X_1_X_3_	1	0.157	0.726	0.098	0.108	0.984	0.047	24.932	0.288	2486.50
X_2_X_3_	1	−0.548	0.295	1.202	3.454	0.554	47.709	−18.884	0.391	1426.46
Lack of fit	3		0.071	23.994		0.466	370.356		0.208	14,379.96
Pure error	2			1.214			192.199			2418.98
SS total	14			103.294			5668.741			68,980.27
Intercept		13.782	0.001 *		111.906	0.002 *		143.883	0.019 *	
R^2^		0.7560			0.9008			0.7565		

df: degrees of freedom; Coef.: regression coefficients; SS: sum of squares; *p*: probability of significance; * statistically significant values (*p* ≤ 0.05).

**Table 3:** Variation and variable levels for stability studies of *Pouteria macrophylla* extracts.

	Levels		
Factors	−1	0	1
X_1_ = time of exposure (min)	60	90	120
X_2_ = temperature of exposure (°C)	30	60	90
X_3_ = concentrations (% *m/v*)	0.5	1.5	2.5

## Data Availability

The data presented in this study are available in the article.

## Sample Availability

Samples of the extracts are available from the authors.
